# Efficacy of Microneedling and CO2 Laser for Acne Scar Remodelling: A Comprehensive Review

**DOI:** 10.7759/cureus.55092

**Published:** 2024-02-27

**Authors:** Soham Meghe, Vikrant Saoji, Bhushan Madke, Adarshlata Singh

**Affiliations:** 1 Dermatology, Jawaharlal Nehru Medical College, Datta Meghe Institute of Higher Education and Research, Wardha, IND

**Keywords:** combination therapy, treatment efficacy, scar remodelling, co2 laser, microneedling, acne scarring

## Abstract

Acne scarring is a prevalent issue affecting millions worldwide, with significant psychological and social implications. Microneedling and CO_2_ laser therapy have emerged as promising modalities for acne scar remodelling. Microneedling induces controlled micro-injuries to stimulate collagen production, while CO_2_ laser therapy precisely ablates scar tissue. This comprehensive review evaluates the efficacy, safety, and comparative benefits of microneedling and CO_2_ laser therapy. Literature synthesis reveals both modalities to improve acne scars, albeit with different mechanisms and risks. Factors influencing treatment selection and the role of combination therapy are discussed. Future directions include optimising protocols and exploring novel techniques. Overall, microneedling and CO_2_ laser therapy offer valuable options for acne scar management, empowering individuals to address the physical and emotional burden of scarring.

## Introduction and background

Acne scarring is a common consequence of severe or persistent acne lesions, affecting millions worldwide [1]. These scars, which can manifest as depressions, pits, or raised bumps on the skin, not only have physical effects but also profound psychological impacts. They can lead to reduced self-esteem, social withdrawal, and even depression in some cases. Understanding the nature and implications of acne scarring is crucial for developing effective treatment strategies [1].

Microneedling and CO_2_ laser therapy are prominent modalities for treating acne scars. Microneedling, or collagen induction therapy, involves using fine needles to create controlled micro-injuries in the skin, stimulating collagen production and promoting scar remodelling [2]. On the other hand, CO_2_ laser therapy utilises a high-energy beam of light to precisely ablate the scar tissue, triggering a healing response and encouraging the growth of new, smoother skin. Both techniques have shown promising results in improving the appearance of acne scars, but they differ in their mechanisms of action and associated risks [3].

This review aims to comprehensively analyse the efficacy of microneedling and CO_2_ laser therapy for acne scar remodelling. By synthesising the existing literature and clinical evidence, we aim to evaluate these treatment modalities' effectiveness, safety, and potential benefits. Additionally, this review seeks to compare the outcomes of microneedling and CO_2_ laser therapy, identify factors influencing treatment selection, and discuss the role of combination therapy approaches. Ultimately, this review aims to guide clinicians and patients in making informed decisions regarding the management of acne scars.

## Review

Understanding acne scarring

Types of Acne Scars

**Figure 1:**
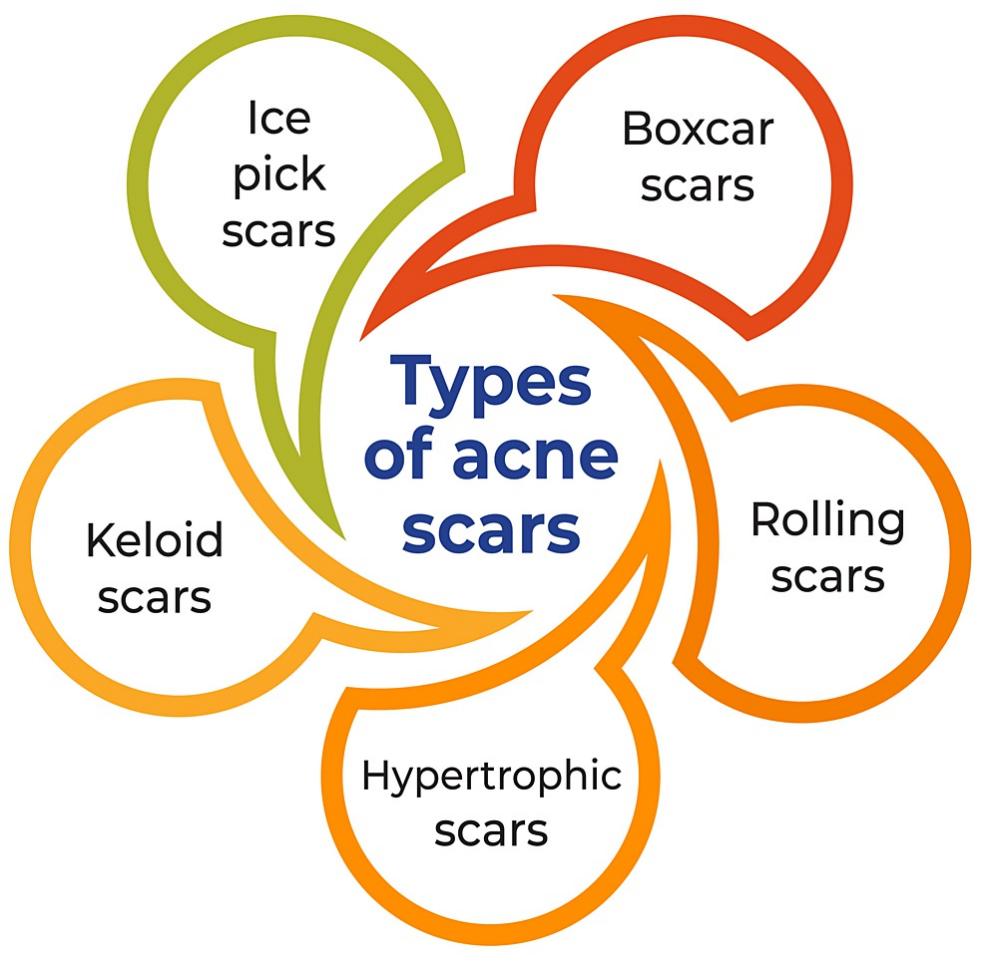
Types of Acne Scars This image has been created by the corresponding author.

Acne scars can manifest in two primary forms: atrophic and hypertrophic scars. Atrophic scars develop due to tissue loss, resulting in indentations in the skin, whereas hypertrophic scars appear raised. The main atrophic scars are ice pick, boxcar, and rolling scars. Ice pick scars are narrow, deep, and V-shaped, whereas boxcar scars are wider with distinct edges, and rolling scars have a sloping edge. Hypertrophic scars and keloids present as raised lumps of scar tissue. The treatment choice for acne scars hinges on size, depth, nature, and location. Various treatments, including chemical peels, dermabrasion, laser treatment, punch techniques, dermal grafting, skin needling, and combined therapies, are available for managing acne scars [4-7]. It is crucial to note that most individuals with acne scars have atrophic scars resulting from collagen loss, and treatment decisions should be tailored to each individual's specific circumstances, considering the scars' size, depth, nature, and location [7]. Types of acne scars are shown in Figure 1.

Factors Influencing Scar Formation

**Figure 2:**
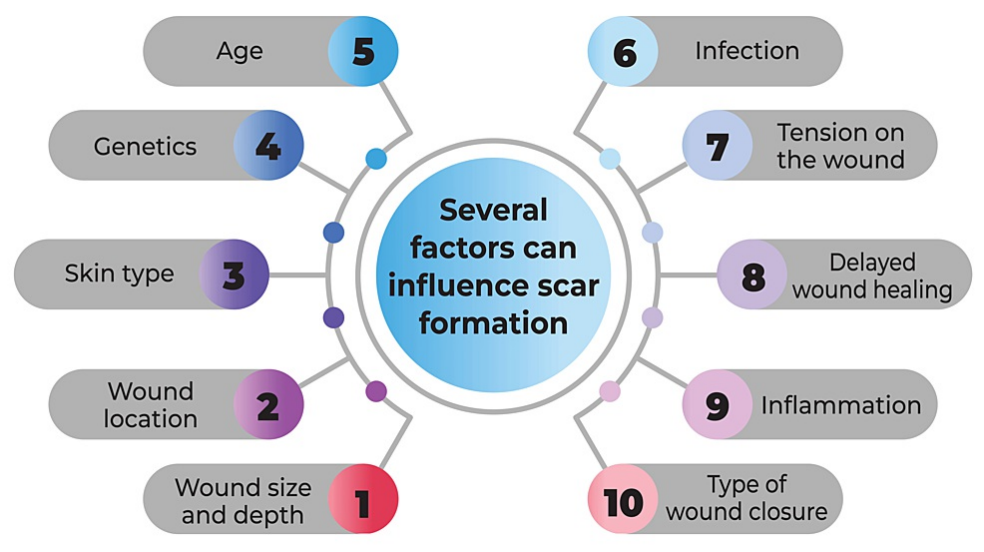
Factors Influencing Scar Formation This image has been created by the corresponding author.

Numerous factors contribute to scar formation, encompassing genetic predispositions, the body's ability to respond to injury, and the intensity and duration of inflammation. Acne scars arise from an aberrant wound-healing response to skin inflammation, with inflammatory cell infiltration observed in 77% of atrophic scars [8]. Despite ongoing research, the precise pathogenesis of acne scarring remains incompletely elucidated, with various hypotheses proposed, including heightened sebum production, alterations in sebum lipid quality, androgen activity, proliferation of *Propionibacterium acnes* (*P. acnes*) within hair follicles, and follicular hyperkeratinisation [4]. Notably, the severity of acne scarring correlates with the grade of acne, underscoring the importance of early intervention in acne lesion management to prevent or mitigate scar development [8]. Additional factors exacerbating scarring encompass habits such as picking or squeezing primary lesions, tobacco use, and delays in initiating acne treatment [7,8]. To determine the most appropriate treatment tailored to individual needs and skin characteristics, it is imperative to seek guidance from a dermatologist. Factors influencing scar formation are shown in Figure 2.

Challenges in Treating Acne Scars

Treating acne scars poses a significant challenge, with a range of available options, including laser therapy, microneedling, chemical peels, and surgical interventions. The selection of treatment modality hinges on the type and severity of the scars. It is crucial to ensure that the skin is clear of blemishes before initiating scar treatment, as medications and treatments used for acne management may interfere with scar treatments. Individuals undergoing scar treatment should anticipate making decisions regarding the type of treatment. They should understand that eliminating scars may only sometimes be achievable, although most treatments can effectively reduce their size and visibility. Given the potential psychological distress caused by acne scars, seeking treatment is paramount. Consulting a dermatologist is the optimal approach, as dermatologists can devise an individualised treatment plan tailored to the specific type of scars and the patient's unique needs [5,9]. Innovations in acne scar revision, encompassing both energy-based and non-energy-based therapies, present novel strategies for scar treatment. These innovations, from laser therapy and radiofrequency devices to chemical peels and surgical interventions, offer diverse options to match the treatment modality to the patient's requirements and the scar's characteristics [10].

Microneedling: mechanism of action and efficacy

Explanation of Microneedling Procedure

Microneedling is a minimally invasive therapeutic technique employed to address various skin concerns, including acne scars, stretch marks, and wrinkles, by inducing controlled skin injury and triggering a cascade of wound-healing processes that stimulate collagen production [11]. The procedure typically commences with a consultation with a healthcare provider, during which the skin is examined, medical history is reviewed, and treatment objectives are discussed. Before the procedure, the skin is cleansed, and a numbing cream or ointment is applied to alleviate discomfort. Subsequently, the healthcare provider utilises either a hand-held roller or an electric tool equipped with tiny needles to create controlled wounds in the skin. The roller or tool is applied slowly and gently across the skin, with the duration varying from 15 minutes to several hours, contingent upon the size of the treatment area [11]. Following the procedure, individuals may experience redness and swelling of the skin for up to five days, although most can resume wearing makeup the day after treatment. While microneedling is generally considered safe, potential risks include infection, scarring, and hyperpigmentation [11].

How Microneedling Affects Acne Scars

Microneedling is an effective treatment for diminishing the visibility of acne scars. Operating on the principle of inducing controlled micro-injuries in the skin, this procedure prompts the production of collagen, thereby facilitating the healing and restructuring of the skin. Its efficacy particularly shines in improving the appearance of depressed acne scars, such as rolling and boxcar scars [12,13]. Moreover, microneedling is hailed for its safety across all skin tones, including darker complexions, as it bypasses damage to the outer layer of the skin [12,13]. The effectiveness of microneedling in addressing acne scars finds robust support from clinical studies, showcasing notable enhancements in skin texture and the reduction of acne scar visibility [2]. Generally well-tolerated, the procedure may elicit minor side effects like temporary redness and bruising, typically subsiding within a few days [12]. However, individuals experiencing an active acne breakout are advised against microneedling, and certain medical conditions or medications may contraindicate the procedure [14]. Overall, microneedling emerges as a promising avenue for individuals seeking to ameliorate the appearance of acne scars, with its efficacy substantiated by both clinical observations and histological evaluations [2].

Review of Clinical Studies Assessing Microneedling Efficacy for Acne Scar Treatment

The efficacy of microneedling in treating acne scars has garnered substantial support from numerous studies. A systematic review encompassing 33 recent peer-reviewed studies revealed consistent improvement in patients' acne scars following microneedling treatment, with many studies reporting high levels of patient satisfaction across various types of microneedling devices [15]. Furthermore, a separate study assessing the clinical impact of microneedling on atrophic acne scars demonstrated that all patients exhibited clinical improvement in scar appearance and skin texture, accompanied by a significant enhancement in overall skin appearance and patient satisfaction [2]. Similarly, research conducted on Vietnamese patients concluded that microneedling represents an effective and safe approach for treating atrophic acne scars, yielding high levels of patient satisfaction without severe complications [16]. While the existing evidence underscores the efficacy of microneedling in acne scar treatment, some studies suggest the need for more direct comparisons with other minimally invasive treatments to elucidate its comparative effectiveness [15] comprehensively. Moreover, it is imperative to recognise that the selection of treatment modality should be individualised based on patient-specific characteristics and preferences.

CO_2_ laser: mechanism of action and efficacy

Explanation of CO_2_ Laser Procedure

Carbon dioxide (CO_2_) laser resurfacing is a skin treatment that harnesses a laser emitting a wavelength of 10,600 nanometres to ablate the superficial layer of the skin, thereby stimulating the generation of new skin and collagen. The CO_2_ laser emits light at a specific wavelength readily absorbed by water in the skin cells, resulting in the precise removal of the superficial skin layer. This process prompts the skin to regenerate new skin and collagen, ultimately rejuvenating the skin [17,18]. During the procedure, the operator and the patient wear laser-safe protective eye equipment to safeguard against potential ocular damage. The operator, often a licensed physician such as a dermatologist or plastic surgeon, utilises the CO_2_ laser to address various skin conditions, including photoaging, wrinkles, and scarring resulting from acne, trauma, or surgical procedures. Laser settings can be tailored to control the depth and extent of the treatment [17,19].

The personnel involved in the procedure typically include the operator, who may be supported by trained assistants such as physician assistants or nurses, who must undergo comprehensive training in laser safety protocols [19]. CO_2_ lasers find utility in treating and preventing photoaging, reducing the appearance of scarring, and managing various skin conditions such as acne scars, fine lines, wrinkles, and skin ageing, with notable efficacy observed in individuals with Fitzpatrick skin types I-II [18,19]. However, the procedure may entail specific side effects, including infection, delayed wound healing, scarring, skin peeling, redness, and alterations in skin tone. Complete skin healing following CO_2_ laser treatment typically requires two to four weeks, during which new skin begins to regenerate around the two-week mark, initially presenting as raw and potentially draining immediately post-procedure. Patients are advised to minimise direct exposure and adhere to proper sun protection measures throughout recovery [18].

How CO_2_ Laser Affects Acne Scars

CO_2_ laser resurfacing emerges as a highly effective treatment for diminishing the visibility of acne scars. Utilising a carbon dioxide laser, this procedure meticulously removes damaged skin layer by layer while stimulating collagen production within the underlying skin. Through this mechanism, scar tissue can be fragmented, paving the way for the emergence of newer, healthier skin, thereby markedly reducing the appearance of acne scars [20]. Notably, fractional CO_2_ lasers have demonstrated significant efficacy in improving most atrophic acne scars, with studies showcasing considerable enhancements in moderate-to-severe acne scars [21]. The treatment is particularly well-suited for addressing superficial to medium-depth scars, encompassing boxcar scars, shallow pick and ice pick scars, superficial rolling scars, and hypertrophic scars [22]. Fractional CO_2_ lasers, administered in a fractional mode, offer exceptional outcomes with minimal downtime, risks, and associated costs [22]. Additionally, a study underscored the substantial improvement achieved in moderate-to-severe acne scars, with fractional CO_2_ lasers yielding a 50% enhancement [23]. Consequently, CO_2_ laser resurfacing emerges as a valuable option for individuals seeking to mitigate the appearance of acne scars.

Review of Clinical Studies Assessing CO_2_ Laser Efficacy for Acne Scar Treatment

Various clinical studies have extensively investigated the efficacy of fractional CO_2_ laser in treating acne scars. While some studies suggest that further evidence is needed to fully establish the clinical efficacy of this treatment modality [24], others provide compelling evidence supporting its effectiveness. A randomised study concluded that fractional CO_2_ laser resurfacing surpasses microneedling and platelet-rich plasma (PRP) efficacy for post-acne scarring [25]. Furthermore, a systematic review and meta-analysis of randomised controlled trials indicated that combining fractional CO_2_ laser therapy with hyaluronic acid dressing is a safe and effective option for treating facial atrophic acne scars [26]. Additionally, a randomised controlled clinical trial found that combining fractional CO_2_ laser with stromal vascular fraction (SVF) yielded superior results compared to fractional CO_2_ laser alone in treating burn scars [27]. These studies provide robust evidence supporting the efficacy of fractional CO_2_ laser therapy for treating acne scars, whether used as a standalone or combined with other modalities.

Comparison of microneedling and CO_2_ laser

Efficacy Comparison Based on Clinical Studies

Several clinical studies have undertaken a comparative analysis of microneedling and CO_2_ laser therapy for treating acne scars, revealing nuanced insights into their respective efficacy and suitability for different patient populations. A randomised study investigating fractional CO_2_ laser, microneedling, and platelet-rich plasma (PRP) in post-acne scarring found that fractional CO_2_ laser exhibited statistically superior therapeutic efficacy compared to microneedling and PRP [25]. Similarly, another study noted that fractional CO_2_ laser demonstrated superior outcomes in scar treatment compared to microneedling, showcasing improvements in texture, pigmentation, contour, brightness, and distortion [23]. However, contrasting findings emerged from a prospective, nonrandomised, open-label study, which concluded that both modalities are equally effective in treating acne scars. Notably, fractional microneedling radiofrequency (MNRF) was identified as having lesser downtime and post-inflammatory hyperpigmentation (PIH) among individuals with darker skin shades, rendering it a more efficient and safer treatment option compared to fractional CO_2_ laser [25]. Additionally, a split-face trial indicated that both methods independently enhanced the appearance of acne scarring, with participants reporting more discomfort during CO_2_ laser treatment [28]. While laser therapy may boast superior efficacy data in comparison to microneedling, it may also be associated with increased adverse events or longer downtime [28]. Consequently, these findings underscore the importance of considering various factors, including downtime, post-inflammatory hyperpigmentation, and individual patient characteristics, when selecting microneedling and CO_2_ laser therapy for acne scar treatment. Ultimately, a tailored approach for these factors is crucial to optimising treatment outcomes and patient satisfaction.

Safety Profile Comparison

Several studies have conducted comparative assessments of the safety profiles of microneedling and CO_2_ laser therapy for acne scar treatment, shedding light on their respective safety profiles and efficacy. One study concluded that microneedling was as safe as fractional CO_2_ laser for rejuvenating traumatic scars, with comparable clinical effects observed [29]. However, another study reported that microneedling boasts an excellent safety profile but is relatively less effective than CO_2_ laser therapy [25]. Adverse effects associated with both treatments, such as crusting and post-inflammatory hyperpigmentation (PIH) in the CO_2_ laser group and bruising and pain in the platelet-rich plasma (PRP) group, were transient and self-limiting [25]. Notably, laser therapy may be linked to increased adverse events or longer downtime compared to microneedling [28]. Overall, findings from these studies underscore the safety of microneedling and CO_2_ laser therapy for acne scar treatment. However, choosing between the two modalities may hinge on individual patient characteristics and preferences. Consulting a dermatologist is paramount to determine the most suitable treatment approach tailored to individual needs and skin characteristics. By considering these factors, clinicians can optimise treatment outcomes and enhance patient satisfaction.

Consideration of Cost-Effectiveness

The cost of microneedling and CO_2_ laser treatments for acne scar removal can exhibit significant variability. According to GoodRx data, laser acne scar removal costs from $200 to $3,000, with average out-of-pocket expenses totalling around $2,000 for ablative and $1,100 for non-ablative laser procedures [30,31]. Specific laser treatments, such as V-Beam, eMatrix, and Fraxel, may incur costs ranging from $800 to $1,500 per session, often necessitating multiple sessions for optimal results [32]. Conversely, microneedling costs upwards of $500 per session, with multiple sessions often required to achieve desired outcomes [32]. It is essential to recognise that these cost estimates are approximate and can vary based on individual needs and the severity of scarring [32]. Moreover, it is crucial to consider the potential trade-offs between cost and treatment efficacy. While laser therapy may demonstrate superior efficacy compared to microneedling, it may also be associated with increased adverse events or longer downtime [29]. Therefore, when evaluating the cost-effectiveness of these treatments, it is essential to weigh the potential benefits against the associated costs and any related downtime or adverse effects. Ultimately, consulting with a dermatologist can provide valuable guidance in selecting the most suitable and cost-effective treatment option tailored to individual needs and preferences.

Combination therapy: Microneedling and CO_2_ laser

Rationale for Combining Treatments

The rationale for combining treatments, such as microneedling and CO_2_ laser therapy, stems from the need to comprehensively address the diverse nature of acne scars and optimise treatment outcomes. Acne scars exhibit morphological variability, with different procedures more suited to specific scar subtypes. For instance, deep ice pick scars may benefit from punch excision or punch graft procedures, while depressed rolling scars may respond well to subcision. Fractional lasers are effective in treating superficial textural irregularities and colour variation. Therefore, combining multiple procedures can lead to enhanced outcomes, particularly when addressing deep scars of varying types and fine textural irregularities [33]. Combining microneedling and CO_2_ laser therapy can result in fewer treatment sessions, reduced costs, and improved patient comfort. Research has shown that this combination can enhance skin texture, stimulate collagen production, and reduce acne scarring. However, it is important to note that the recovery time may be longer depending on the sensitivity of the treated area after the procedure [34]. Moreover, a combination of procedures, such as chemical reconstruction of skin scars (CROSS), subcision, and microneedling, has demonstrated effectiveness in treating acne scars, with consistently high satisfaction reported by patients and photographic evidence of improvement [35]. This underscores the potential synergistic effects of combining different treatment modalities to achieve optimal results in acne scar management.

Clinical Evidence Supporting Combination Therapy

Combination therapy involving microneedling and CO_2_ laser has emerged as a highly effective approach for enhancing skin texture, reducing acne scarring, and promoting collagen production [23,34,36]. Evidence from a randomised, single-treatment, split-face trial comparing fractional CO_2_ laser and microneedle radiofrequency for acne scars indicated that each method independently improved the appearance of acne scarring, albeit with reported discomfort during treatment. Notably, the trial did not explore combining both treatments [37]. While microneedling and CO_2_ laser therapy offer distinct benefits, their combined approach can yield superior outcomes for patients grappling with various skin concerns within a single procedure. Consulting with a dermatologist or surgeon is crucial to ensure the safety of combined procedures and to formulate the most suitable treatment plan tailored to individual needs and medical history. By leveraging the synergistic effects of microneedling and CO_2_ laser therapy, patients can achieve optimal results in addressing acne scarring and enhancing overall skin quality.

Advantages and Disadvantages of Combination Therapy

Combination therapy, such as fixed-dose combination, presents both advantages and disadvantages. Some advantages encompass enhanced compliance, synergy, increased efficacy, reduced side effects, and cost-effectiveness [38,39]. Conversely, potential drawbacks of combination therapy may entail an inflexible fixed dose ratio, incompatible pharmacokinetics, heightened toxicity, and the necessity for meticulous consideration of the specific circumstances in which combinations are employed [38,40]. Moreover, concerns may arise regarding the loss of dose flexibility and the potential for increased cost in certain instances [40]. Healthcare providers must meticulously assess each patient's medical needs and circumstances to weigh the benefits and drawbacks of combination therapy carefully.

Patient selection and considerations

Factors Influencing Treatment Choice

When deciding on a treatment for acne scars, several factors merit consideration, including skin type, scar type, and patient preference. Fractional CO_2_ laser and microneedling are effective treatments for acne scars. Still, the optimal choice between the two may hinge on factors such as skin type, tolerability, and safety profile. A randomised study comparing fractional CO_2_ laser, microneedling, and platelet-rich plasma (PRP) in post-acne scarring concluded that fractional CO_2_ laser exhibited statistically superior therapeutic efficacy compared to microneedling and PRP [37]. Conversely, another study comparing the efficacy, safety, and tolerability of fractional CO_2_ laser versus fractional microneedling radiofrequency (MNRF) in acne scars found that MNRF was deemed more efficient, better tolerated, and comparatively safer, particularly for patients with darker skin tones [25]. Furthermore, a split-face trial comparing fractional CO_2_ laser and microneedle radiofrequency for acne scars revealed that each method independently enhanced the appearance of acne scarring, with participants reporting more significant discomfort during treatment [23]. Additional considerations include the patient's medical history, the severity of the scarring, and the patient's treatment expectations. Consulting a dermatologist is crucial to determine the most appropriate treatment based on individual needs and skin characteristics. By carefully assessing these factors, clinicians can tailor treatment plans to optimise outcomes and ensure patient satisfaction.

Patient Demographics Suitable for Each Treatment

The choice between fractional CO_2_ laser and microneedle radiofrequency for treating acne scars hinges on various factors, including patient demographics and skin characteristics. Evidence suggests that microneedling may be more appropriate for individuals with darker skin tones, as it is deemed more effective, better tolerated, and relatively safer for this demographic [41]. Conversely, the fractional CO_2_ laser has demonstrated greater efficacy in patients with atrophic acne scars, with the potential for notable improvement in certain scar types, albeit with a heightened risk of post-inflammatory hyperpigmentation in individuals with darker skin tones [41]. Furthermore, a randomised study underscored the superior therapeutic efficacy of fractional CO_2_ laser compared to microneedling in post-acne scarring [25]. Given the nuanced considerations of patient demographics, skin type, and specific acne scar characteristics, a dermatologist must seek guidance when selecting the most appropriate treatment approach for each individual. Through a comprehensive evaluation, dermatologists can tailor treatment plans to optimise outcomes while mitigating potential risks and complications, ensuring the best possible results for patients.

Potential Contraindications and Precautions

Careful patient selection is paramount when contemplating microneedling and CO_2_ laser for acne scar remodelling. Individuals with darker skin types may face an elevated risk of post-inflammatory hyperpigmentation (PIH). They, thus, should undergo priming with chemical peels and laser toning before receiving ablative fractional CO_2_ laser treatment to diminish recent tan and pigment at the scar base [41]. Patients with a history of keloid or hypertrophic scarring may not be ideal candidates for these treatments. Furthermore, individuals with active acne or infections in the treatment area should postpone treatment until the condition has resolved. Patients must also be educated about potential side effects, including erythema, PIH, and procedural discomfort, and adhere meticulously to post-treatment care instructions [8]. Consulting a dermatologist is crucial to determine the most appropriate treatment plan tailored to individual needs and skin characteristics. Dermatologists can ensure optimal treatment outcomes through personalised evaluation and guidance while prioritising patient safety and satisfaction.

## Conclusions

In conclusion, the efficacy of microneedling and CO_2_ laser therapy for acne scar remodelling has been extensively studied and documented. Both modalities have shown considerable promise in improving the appearance of acne scars and the overall quality of life for affected individuals. Microneedling offers a non-invasive approach that stimulates collagen production and promotes scar remodelling, while CO_2_ laser therapy provides a precise ablation of scar tissue and encourages skin regeneration. However, it is essential to recognise that the selection of treatment modality should be based on various factors, including scar type, severity, patient preferences, and skin type. Moreover, combination therapies involving microneedling and CO_2_ laser or other modalities may offer synergistic benefits and improved outcomes in some instances. Future research efforts should focus on optimising treatment protocols, identifying predictors of treatment response, and exploring novel techniques for acne scar management. Additionally, continued education and training for healthcare providers are essential to ensure the safe and effective delivery of these treatments. Overall, microneedling and CO_2_ laser therapy represent valuable options for individuals seeking to address the physical and psychological burden of acne scarring. By understanding the strengths and limitations of each modality, clinicians can tailor treatment plans to meet the unique needs of their patients, ultimately fostering greater satisfaction and confidence in those affected by acne scars.
